# Genomic and epidemiological evidence for the emergence of a *L. infantum/L. donovani* hybrid with unusual epidemiology in northern Italy

**DOI:** 10.1128/mbio.00995-24

**Published:** 2024-06-04

**Authors:** F. Bruno, G. Castelli, B. Li, S. Reale, E. Carra, F. Vitale, S. Scibetta, M. Calzolari, S. Varani, M. Ortalli, E. Franceschini, W. Gennari, G. Rugna, G. F. Späth

**Affiliations:** 1 WOAH Leishmania Reference Laboratory, Istituto Zooprofilattico Sperimentale della Sicilia, Centro di Referenza Nazionale per le Leishmaniosi (C.Re.Na.L.), Palermo, Italy; 2 Bioinformatics and Biostatistics Hub, Institut Pasteur, Université Paris Cité, Paris, France; 3 Istituto Zooprofilattico Sperimentale della Lombardia e dell'Emilia Romagna "B. Ubertini", Brescia, Italy; 4 Department of Medical and Surgical Sciences, University of Bologna, Bologna, Italy; 5 IRCCS Azienda Ospedaliero-Universitaria di Bologna, Bologna, Italy; 6 Infectious Disease Unit, Azienda Ospedaliera Universitaria di Modena, Modena, Italy; 7 Virology and Molecular Microbiology Unit, University Hospital of Modena, Modena, Italy; 8 Unité de Parasitologie moléculaire et Signalisation, INSERM U1201, Institut Pasteur, Université Paris Cité, Paris, France; Washington University in St. Louis School of Medicine, St. Louis, Missouri, USA; NIH, NIAID, Bethesda, Maryland, USA

**Keywords:** *Leishmania*, comparative genomics, genetic hybrid, visceral leishmaniasis

## Abstract

*Leishmania* (*L*.) *infantum* is one of the main causative agents of animal and human leishmaniasis across many endemic areas in South America, Europe, North Africa, and Asia. Despite its clinical significance, little is known about the genetic diversity of *L. infantum* circulating in a given endemic area. Here, we investigate this important open question by applying a comparative genomics approach to seven *L. infantum* isolates from different hosts and Italian regions, including the northern part of the country (Emilia-Romagna, RER), Sicily, and Sardinia, as an initial attempt to explore the breadth of parasite genetic heterogeneity in Italy. Additionally, microsatellite analysis was carried out to compare the isolates from RER with other 70 *L*. *infantum* strains from the same region as well as 65 strains belonging to the *L. donovani* complex from other countries. We revealed important karyotypic instability and identified strain-specific changes in gene dosage, which affected important virulence factors such as amastins and surface antigen-like proteins. Single nucleotide polymorphism-based clustering analysis of these genomes together with over 80 publicly available *L. infantum* and *L. donovani* genomes placed the Italian isolates into three geographically distinct clusters within the Mediterranean basin and uncovered three isolates clustering with putative *L. infantum/L. donovani* hybrids isolated in Cyprus. As judged by microsatellite profiling, these hybrid isolates are representative of a sub-population of parasites circulating in northern Italy that preferentially infect humans but not dogs. Our results place Italy at the crossroads of *L. infantum* infection in the Mediterranean and call attention to the public health risk represented by the introduction of non-European *Leishmania* species.

**IMPORTANCE:**

This study closes important knowledge gaps with respect to *Leishmania (L.) infantum* genetic heterogeneity in a given endemic country, as exemplified here for Italy, and reveals genetic hybridization as a main cause for re-emerging human leishmaniasis in northern Italy. The observed high diversity of *Leishmania* parasites on the Italian peninsula suggests different geographical origins, with genomic adaptation to various ecologies affecting both pathogenicity and transmission potential. This is documented by the discovery of a putative *L. infantum*/*L. donovani* hybrid strain, which has been shown to preferentially infect humans but not dogs. Our results provide important information to health authorities, which need to consider the public health risk represented by the introduction of new *Leishmania* species into EU countries due to population displacement or travel from countries where exotic/allochthonous parasite species are endemic.

## INTRODUCTION

Leishmaniases are a group of sand fly-transmitted parasitic diseases that can affect mammals, including humans. In humans, these diseases are characterized by cutaneous, muco-cutaneous, or visceral tissue damage, the latter often being fatal if left untreated. The pathogenesis and clinical outcome of *Leishmania* infection rely on a series of parasite-specific traits that ensure proliferation and fitness gain in different host systems.

First, these parasites have evolved different life cycle stages, including motile promastigote forms that are adapted for survival inside the midgut of phlebotomine sand flies and intracellular amastigotes that infect immune cells of the reticuloendothelial system inside the mammalian host. Second, aside stage differentiation, *Leishmania* can further adapt to environmental variations encountered inside both insect and vertebrate hosts; this phenomenon has been linked to the intrinsic plasticity of the *Leishmania* genome and the genetic recombination that can follow the formation of parasite hybrids between strains and species inside the insect vector. In the wake of next-generation sequencing and the development of powerful computational pipelines for sequencing data analysis (1), comparative genomics approaches have provided important insight into the epidemiology of *Leishmania* infection in the field or parasite fitness gain in experimental settings. For example, comparative genomics has informed on (i) the mechanisms underlying drug resistance and parasite evolution in treated patients (2), (ii) the origin, population structure, and phylogenetic relationship of clinical isolates, (iii) hybridization events and their effect on tissue tropism or clinical outcome of infection (3, 4), (iv) the role of dynamic gene dosage changes in parasite fitness gain (5), or (v) the genomics of geographic adaptation, which is the scope of the current study.

*Leishmania infantum* causes leishmaniasis in Mediterranean Europe, with significant emergence of human cases observed in localized areas of Spain (6) and Italy (7). Italy is hypo-endemic for human leishmaniasis, with less than 100 cases of cutaneous and visceral leishmaniasis (VL) reported to the WHO in 2020 (8), while dogs are considered the main reservoir hosts. Historically, leishmaniasis in Italy was restricted to the Tyrrhenian littoral, the southern peninsula, and the islands. However, in the last 20 years, sand fly vectors as well as human and canine *Leishmania* infections have been detected in northern Italy, traditionally classified as a cold area unsuitable for sand fly survival (9).

In the last decade, a surge of human leishmaniasis cases has been observed in the Emilia-Romagna region (RER), northern Italy (7). However, there was no concurrent increase in the prevalence of canine leishmaniasis in the same period. In addition, molecular typing studies in RER showed that the *Leishmania* strains circulating in dogs belonged to a different population compared to strains isolated from human VL cases and sand flies (10). Furthermore, the biting preference of *Phlebotomus (Ph) perfiliewi*, the suspected vector responsible for the increase of VL cases in RER, suggested the presence of a peri-urban or sylvatic reservoir other than dogs, which is capable of spreading the infection in such areas (11). Consistent with this observation, a high frequency of *Leishmania* infection was recently identified in other potential reservoir hosts, including roe deer, hares, wolves, or red foxes (12).

While some studies have evaluated the epidemiology of VL in Italy, genetic characterization of *L. infantum* across different regions is lacking. Here, we combined comparative genomics and microsatellite profiling approaches on *L. infantum* field isolates that were obtained in Italy to shed light on parasite genetic heterogeneity. We provide evidence for a remarkable genetic diversity of *L. infantum* across the Italian peninsula and identify the strains from RER as putative *L. infantum/L. donovani* hybrids, which are closely related to Cypriot strains, thus correlating their unusual epidemiology and transmission pattern to their unique genetic constitution.

## RESULTS

### Cultured *L. infantum* isolates show strain-specific and convergent aneuploidies

**Fig 1:**
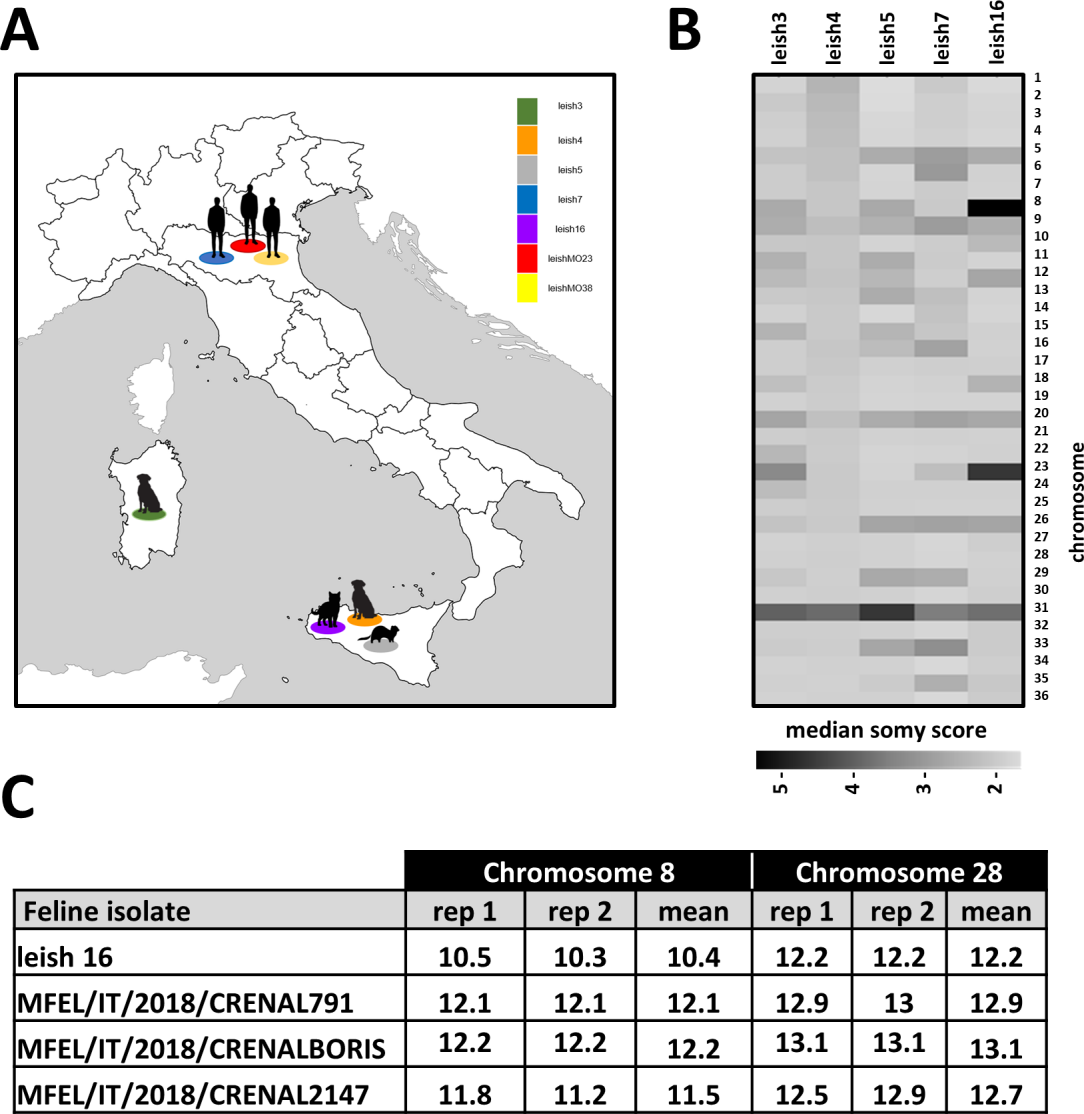
Karyotypic analyses of the *L. infantum* isolates (*n* = 5). (**A**) Map indicating the geographic location and origin of the *L. infantum* isolates investigated in this study. (**B**) Heatmap showing the median somy score for the different strains across the 36 chromosomes. Darker tonalities reflect higher somy values. (**C**) Quantitative PCR analysis with SYBR Green assay to quantify chr 8 copy number in feline *Leishmania* strains. CT values are shown for the histone deacetylase gene on chr 8, and the glycerophosphoryl diester phosphodiesterase gene on disomic chr 28 used as a control.

We applied a comparative genomics approach to gain insight into the genetic diversity of *L. infantum* field isolates in Italy. We deliberately chose initially five isolates from different hosts (human, dog, cat, and marten) and geographic regions (RER in northern Italy, Sicily, and Sardinia) to maximize the assessment of parasite genetic heterogeneity (Table 1; Fig. 1A). Following culture expansion, DNA extraction, and Illumina short read sequencing, we applied our genome instability pipeline (GIP) (1) to map the reads on the JPCM5 *L. infantum* reference genome (13) and assess read depth variations for each individual sample. We then compared chromosome read depth variations between samples using per chromosome boxplots of normalized coverage (somy score) in 300 bp genomic bins (see Materials and Methods). As judged by the changes in the somy score, all samples showed important karyotypic variations, thus confirming the intrinsic instability of the *L. infantum* genome as previously reported (2) (Fig. S1). As expected from previous studies, all samples were tetrasomic for chromosome (chr) 31 (14) while displaying unique karyotypic profiles considering the other chromosomes. This diversity may be largely attributed to the strain-specific selection of different aneuploidies during culture adaptation, which we previously linked to *in vitro* fitness gain (5). The heat map shown in Fig. 1B reveals leish4, a strain isolated from a Sicilian dog from Palermo (Table 1), as the karyotypically most stable sample, with most chromosomes showing a median somy score between 2 and 2.5. All other samples showed more important somy variations, including full trisomies or higher, mosaic aneuplodies.

**TABLE 1:** Overview of the *L. infantum* isolates that were analyzed in this study by whole-genome sequencing

Lab ID	International code	Host/location (province)/date/ culture passage (p)	Information	Sequencing reads: number/% mapped
leish3	MCAN /IT/2021/51327	Dog/Sardinia (Cagliari)/2021/p8	Glucantime resistant	27,739,245/0.98
leish4	MCAN/IT/2020/1265	Dog/Sicily (Palermo)/2020/p8	–^*a*^	25,856,894/0.91
leish5	MMST/IT/2006/V2921	Marten/Sicily (Palermo)/2006/p15	–	21,183,149/0.98
leish7	MHOM/IT/2014/IZSLER-MO22	Human/Emilia-Romagna (Modena)/2014/p8	VL case; comorbidity: solid neoplasia	26,421,239/0.98
leish16	MFEL/IT/2018/10816	Cat/Sicily (Palermo)/2 018/p16	–	25,578,672/0.81
leishMO23	MHOM/IT/2014/IZSLER-MO23	Human/Emilia-Romagna (Modena)/2014/p8	VL case; comorbidity: hematologic disorder	22,041,069/0.98
leishMO38	MHOM/IT/2016/IZSLER-MO38	Human/Emilia-Romagna (Bologna)/2016/p8	VL case; comorbidity: HIV positive	20,902,208/0.92

^*a*^ "–" indicates that information is not available.

Our data revealed pentasomies for chr 8 and 23 in the leish16 sample. Given that this sample represents the first *L. infantum* genome sequenced from a feline isolate, we investigated if this unusual amplification pattern may be selected in this particular host. This possibility was ruled out by the quantification of chr 8 copy number by quantitative PCR (qPCR) analysis of three independent feline isolates, which showed a disomic amplification score comparable to disomic chr 28 included as a control (Fig. 1C). Thus, the pentasomy for chr 8 may either have been uniquely selected in leish16 during feline infection or underwent important amplification during the 16 culture passages of this sample (see Table 1). Of note, amplification of chr 8 and 23 is quite common in cultured promastigotes of other species. They have been described as among the most frequent polysomal chromosomes in *L. donovani* isolates (15) and have been shown to arise during *L. major* self-hybridization (16).

Thus, the Italian *L. infantum* field isolates show important somy variations in culture compared to the haploid reference genome. Convergent amplification of a small number of chromosomes in most or all strains suggests that these aneuploidies are a potential driver of *L. infantum* fitness gain in culture. Surprisingly, similar chromosomes were linked to culture adaptation of *L. donovani* isolates from the Indian sub-continent and Sudan (e.g., chr 5, 20, and 26) (5), suggesting conserved fitness mechanisms between both parasite species when selected for growth in culture. Aside from these convergent aneuploidies, we further observed strain-specific, karyotypic changes that may fine-tune adaptation in response to other genetic differences between the strains, which we further analyzed in the following section.

### Gene copy number variations in the *L. infantum* isolates affect known *Leishmania* virulence genes

**Fig 2:**
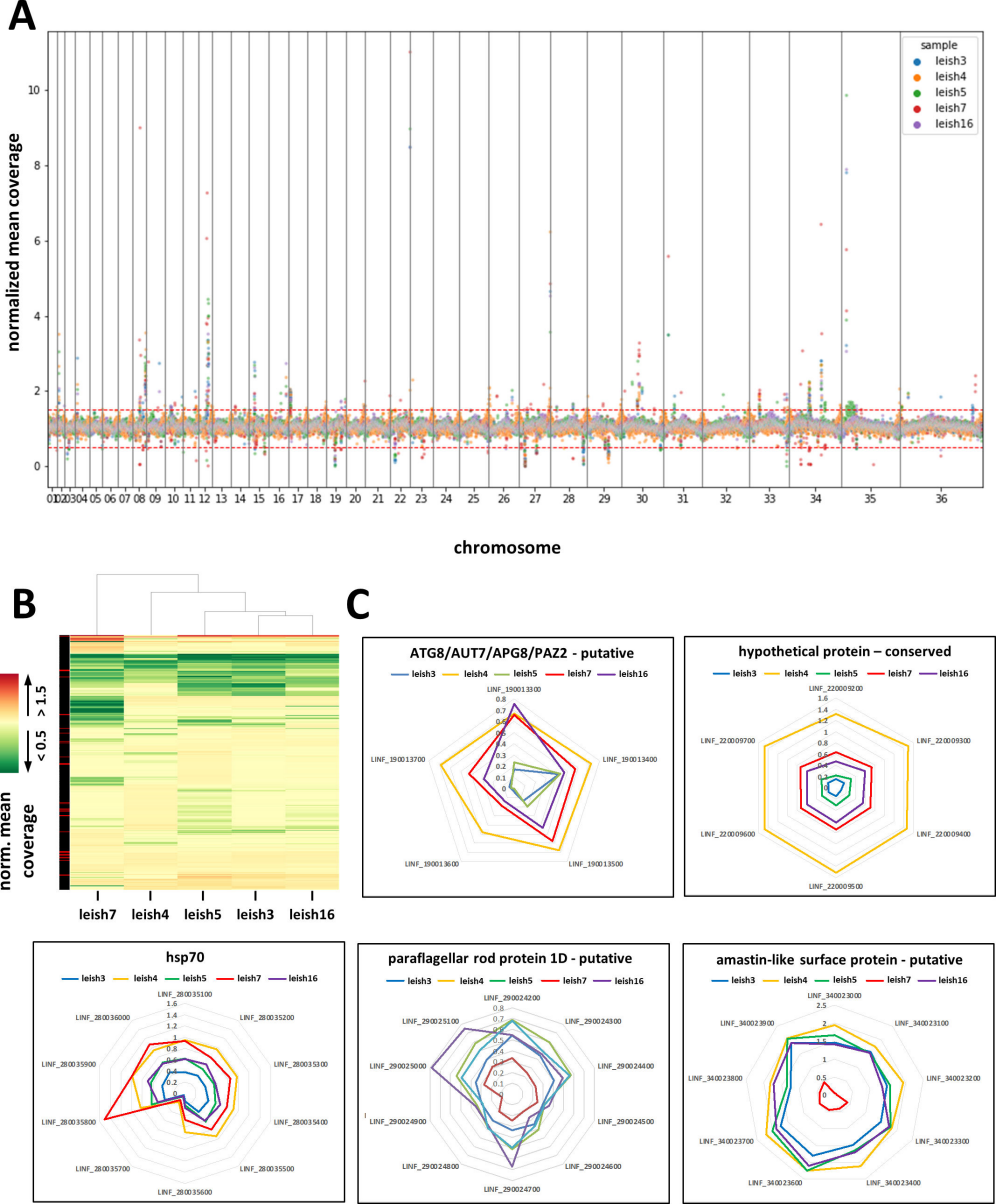
Gene copy number analyses of the *L. infantum* isolates (*n* = 5). (**A**) Scatterplot showing the normalized mean coverage (*y*-axis) for each gene across the 36 chromosomes (*x*-axis). Different strains are shown in different colors (see legend in the figure). (**B**) Heatmap of normalized mean coverage. The plot shows gene depletions defined by a normalized mean coverage < 0.5 (green, dark green corresponding to deletions with read depth = 0) and gene amplifications defined by a normalized mean coverage > 1.5 (red). The column on the left indicates the MAPQ score (red, MAPQ < 40; black, MAPQ > 40). (**C**) Radar plots of the normalized mean coverage values for the genes indicated. Different strains are shown in different colors (see legend in the figure). The expected normalized coverage with respect to the reference genome is 1.

We next examined the gene-level coverage information generated by GIP to assess strain-specific and convergent gene dosage changes with respect to the JPCM5 reference genome (supplemental material; Fig. S2; and Data Set S1A). Unlike karyotypic changes that are rapidly selected during short-term culture adaptation (5–20 passages), gene copy number variations (CNVs) are much less dynamic and thus can reveal gene dosage changes that were selected in the field. Plotting the normalized read depth coverage per gene, we observed a wavy pattern for all isolates caused by minor read depth variations across the chromosomes (Fig. 2A; supplemental material; Fig. S2). A series of gene CNVs were observed, which correspond to the amplification or depletion of gene copies with respect to the reference genome, but also in between the five samples. Significantly, these variations were not scattered across the genome but localized in defined genomic regions that were similar in all samples, thus revealing possible hot spots of gene dosage changes (Fig. 2A). Closer inspection of the 50 most significant gene CNVs identified both single gene and sub-chromosomal amplifications, many of which have been previously linked to infectivity, including amastins (17), surface antigen-like proteins (18), or genes implicated in the synthesis of phosphoglycan virulence factors (19), such as phosphoglycan beta 1–3 galactosyltransferase genes (LINF_020006900 and LINF_020007000) or ppg3 (LINF_350010100 and LINF_350010200) encoding filamentous proteophosphoglycan (20) (supplemental material; Fig. S3A; Data Set S1B). Significantly, most gene amplifications are observed across several isolates. Such convergence is not only observed at the gene level but also at the level of gene function, with amplified genes often sharing the same annotation even though they are on different chromosomes and encoding different proteins, as shown, for example, for the genes encoding surface antigen proteins and amastins (supplemental material; Fig. S3B).

We also identified 116 gene depletions (normalized mean coverage < 0.5) and deletions (normalized mean coverage = 0) that were shared or strain-specific in the five *L. infantum* isolates (Fig. 2B). Most of these changes occurred in multi-copy gene arrays (Data Set S1C), where recombination events between identical sequences allow for dynamic gene copy number changes. In particular, such dynamic variations between the strains were observed for (i) five gene copies on chr 9 encoding for the autophagy gene ATG8, which has been implicated in *Leishmania* stress response and infectivity (21), (ii) six gene copies on chr 22 encoding for an uncharacterized, hypothetical protein, (iii) a cluster of nine amastin genes encoded on chr 34 corresponding to two multi-copy gene arrays (Fig. S3B) and that were previously classified as *δ*-amastins by Jackson (22) known to control amastigote infectivity (17), (iv) 10 HSP70 gene copies encoded on chr 28 that we previously revealed as a potential hot spot of environment-genotype interaction in *L. donovani* field isolates (23), and (v) 10 genes on chr 34 encoding for paraflagellar rod protein (Fig. 2C).

The reduced read depth that we observed for individual gene copies could indicate mosaic gene CNVs, i.e., mixed populations of wild-type and deleted genotypes, or represent a populating-wide, heterozygous state. Only a few genes show true deletions as judged by the absence of reads, including LINF_270010900 likely encoding for a calpain-like protease as suggested by a BLAST search (data not shown), which is deleted in leish16, leish3, leish5, and leish7, or the ATG8 gene LINF_190013600 that is deleted in leish5 (Fig. 2C). The presence of these deletions within non-clonal, heterogeneous parasite populations suggests selection against these genes, even though loss due to genetic drift cannot be ruled out.

### Discovery of leish7 as a highly divergent *L. infantum* strain

**Fig 3:**
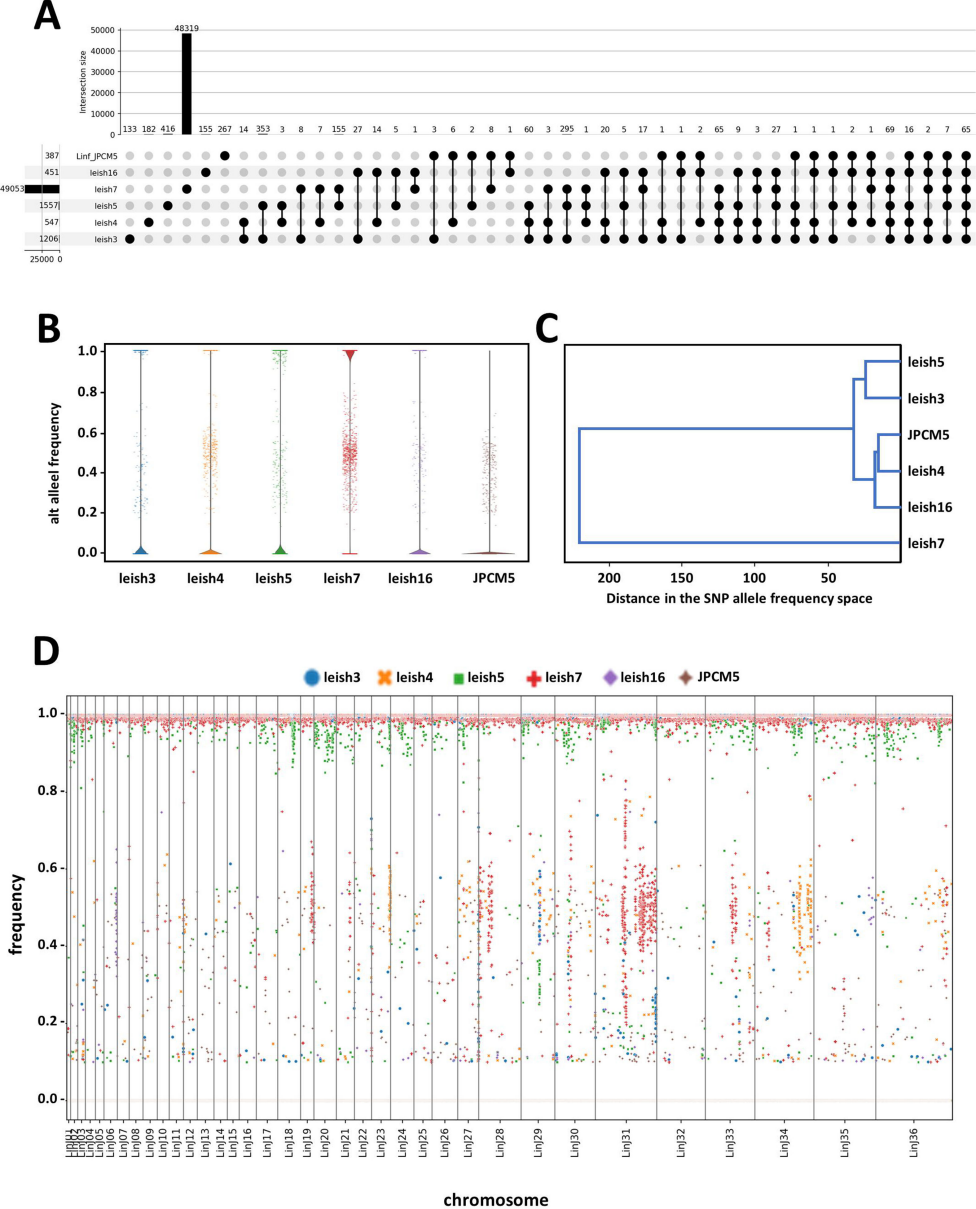
Genome-wide SNP analyses of the *L. infantum* isolates (*n* = 5). (**A**) Upset plot of SNPs identified in the different strains. The bars visualize the number of SNPs common to a given combination of samples. The numbers in the bar plot indicate shared SNPs for the given combination. The numbers on the left side of the strain ID indicate the total number of SNPs in each isolate. (**B**) Violin plot representing density estimates of the distribution of alt-allele frequencies for the indicated samples. Strip plots are superimposed on the violin plots in order to show individual loci at intermediate frequencies. Only those loci where at least one sample had an alt-allele frequency between 0.2 and 0.8 are represented. (**C**) Cluster analysis based on pairwise Euclidean distances between alt-allele frequencies across SNPs. The tree is constructed from these distances using the UPGMA method. (**D**) The scatterplots display individual SNPs as dots. The *x*-axis indicates the relative position of each SNP along the genome, while the *y*-axis indicates the variant allele frequency (see Fig. S4 for individual panels). The different chromosomes are displayed one after the other, and their boundaries are visualized as vertical lines. The individual SNPs are colored according to the samples as indicated by the legend in the graph. The dense superposition of dots corresponding to SNPs at frequency 0 or 1 results in blurry lines at *y* = 0 and *y* = 1. There are no SNP in the (0, 0.1) frequency interval due to the filtering applied during the SNP calling step of the GIP pipeline (the corresponding frequency is set to 0 when data pertaining to various samples are merged together).

We next assessed the evolutionary relationship between our five *L. infantum* isolates by comparing single nucleotide polymorphism (SNP) numbers, positions, and frequencies from the GIP output. Based on the number of alternative (alt) alleles, leish16 is the most similar to the JPCM5 reference genome with only 451 SNPs identified, while leish7 is the most divergent with 49,053 SNPs (Fig. 3A and B; Data Set S1D). Of note, including Illumina reads of the JPCM5 reference strain itself (24) revealed a total of 387 SNPs, of which 267 were unique, while 120 were shared with one or more of the isolates. Cluster analysis based on these variants revealed the highest similarities between JPCM5, leish4, and leish16 on one hand, and leish3 and leish5 on the other hand, while leish7 represented its own branch, with similar distance from either of the two other clusters (Fig. 3C). Plotting frequency against location revealed defined patches of heterozygous SNPs at a frequency of about 50% in leish7, suggesting the presence of distinct haplotypes that may be remnants of former hybridization events (Fig. 3D, see also individual plots shown in Fig. S4).

### Investigating the possible origin of the leish7 putative hybrid

**Fig 4:**
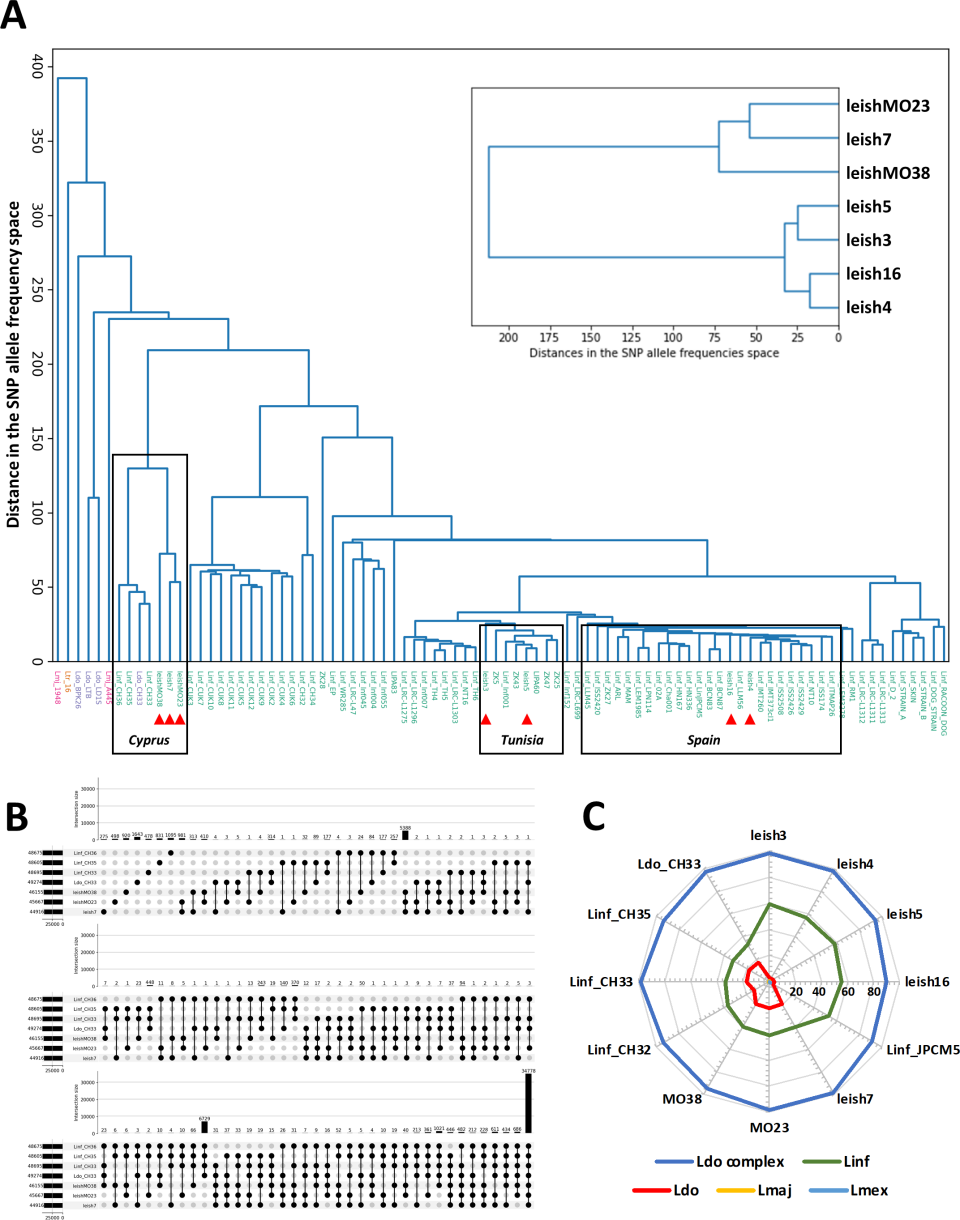
Putative origin of the leish7 hybrid based on SNP analysis. (**A**) Cluster analysis based on pairwise Euclidean distances between alt-allele frequencies across SNPs. The tree is constructed from these distances using the UPGMA method. On the main tree, sample names are colored according to their recorded species. The samples that were not sequenced in the present study have their names prefixed by a tag indicating the species: Lmj (*major*), Ltr (*tropica*), Ldo (*donovani*), or Linf (*infantum*). The samples that were sequenced in the present study are indicated by a red triangle. The top right inset shows the cluster analysis of two additional hybrid strains (leishMO23 and leishMO38) in comparison to the other strains sequenced in this study. (**B**) Upset plot of SNPs identified in the different strains indicated. The bars visualize the number of SNPs common to a given combination of samples. The numbers in the bar plot indicate shared SNPs for the given combination. The numbers on the left side of the strain ID indicate the total number of SNPs in a given isolate. Note that the upset plot continues in the lower panels. (**C**) Radar plot summarizing the results of the Kraken analysis. Along each radial axis, the distance from the center indicates the percentage of reads unambiguously assigned to a given taxon (color encoded) for the corresponding sample. Notable amounts of reads assigned to *L. donovani* (red) are only observed in the (putative) hybrids.

We next established the genome sequence for two additional isolates (leishMO23 and leishMO38) obtained from human VL cases from RER, which have been previously shown to cluster with leish7 (originally named MO22) (10). Cluster analyses revealed their close relationship with leish7, confirming the transmission of a distinct parasite sub-population—likely a hybrid—in RER (Fig. 4A, top right inset, and Data Set S1D). We further expanded the cluster analysis including 11 genomes that we previously generated as part of the LeiSHield project (www.leishield.org) (2) and 63 genomes analyzed by Franssen et al. (15). While four of our Italian *L. infantum* strains clustered with isolates from Tunisia (leish3 and leish5) and Spain (leish4 and leish16), the three RER hybrid-like strains (leish7, leishMO23, and leishMO38) clustered with the known *L. infantum/L. donovani* hybrids first described in Cyprus (Linf_CH33, 35, 36 and Ldo_CH33). Comparison of SNPs between these hybrids revealed over 34,000 shared SNPs (Fig. 4B), indicating that these geographically distinct hybrids are likely the result of similar hybridization events between *L. infantum* and *L. donovani* parental strains. This common hybrid nature is further supported using the Kraken software, a sequence classification tool developed for metagenomic analyses, which assigns taxonomic labels to DNA sequences using the exact alignment of *k*-mers (25). This approach allowed us to classify all strains (including the Italian and Cypriot hybrids) as belonging to the *L. donovani* complex, with only the hybrids revealing a significant match at the species level to *L. donovani* genomes (Fig. 4C). However, whether the RER and Cypriot hybrids are of common origin remains to be established.

### Population structure and phylogenetic analysis of putative hybrid strains from northern Italy

**Fig 5:**
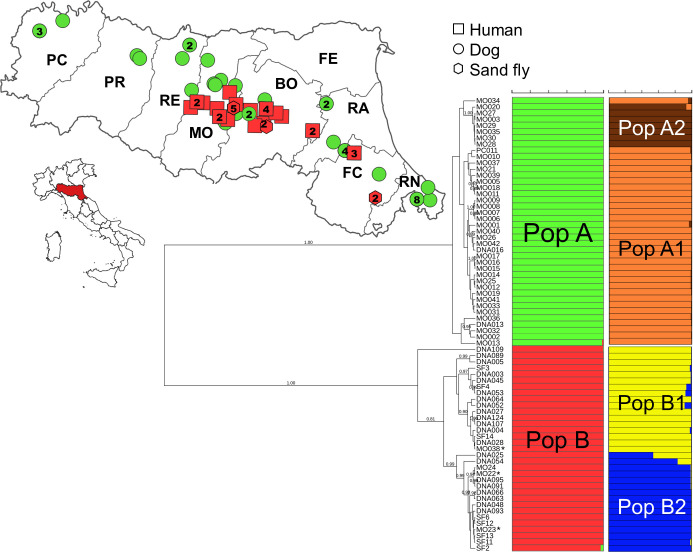
Combined population structure and genetic distance analyses and geographical distribution of populations using the data of 15 microsatellite loci for the 73 *L*. *infantum* strains from the Emilia-Romagna region (northern Italy). (Bottom right) Bayesian phylogenetic tree obtained by the Sainudiin model. Posterior probability values > 0.80 are indicated at the nodes. Populations as inferred by STRUCTURE are indicated by colored bars: green for PopA and red for PopB. The isolates that were sequenced in this study (leish7, leishMO23, and leishMO38) are indicated with an asterisk. (Top left) Geographical distribution of human, canine, and sand fly *Leishmania*-positive samples, 2013–2021, Emilia-Romagna region (northern Italy). The number of strains isolated in a given area are indicated inside the icons (no number=1). Map generated with Quantum-GIS (https://www.qgis.org/it/site/).

In order to better define the prevalence of the leish7 hybrid-like genotype in northern Italy, microsatellite analysis was performed on 73 *L*. *infantum* strains, including 22 strains obtained from human VL cases, 8 strains obtained from sand flies, and 40 strains obtained from canine cases from various areas of RER (Fig. 5). Bayesian clustering as implemented in STRUCTURE assigned the 73 multilocus microsatellite typing (MLMT) profiles from these *L. infantum* strains to two main populations (the highest value of Δ*K* was for *K* = 2; Fig. S5), namely, population A (PopA) that consisted of canine *L. infantum* strains only and population B (PopB) that consisted of all the *L. infantum* strains from VL (including leish7, leishMO23, and leishMO38) and sand flies (Fig. 5). A minor Δ*K* peak at *K* = 5 (Fig. S5) indicated a substructure in the whole data set, which was first explored by increasing the number of populations from *K* = 3 to *K* = 5: PopA exhibited subdivision into two groups at *K* = 3 and three groups at *K* = 4, while PopB split into two groups at *K* = 5 (Fig. S6). Subsequently, we analyzed each of the two main populations (PopA and PopB) separately; the calculation of Δ*K* values (Fig. 5) showed the presence of four subpopulations: PopA1, PopA2, PopB1 (including leishMO38), and PopB2 (including leish7 and leishMO23), while a fifth sub-cluster that was indicated by the minor peak at *K* = 5 in the first STRUCTURE analysis (all strains) was not confirmed.

The spatial distribution of the genetic groups showed that the two main populations (PopA and PopB) overlapped (Fig. 5), while partial overlap was observed for the four subpopulations: PopA1 was widespread all over RER, PopB1 was prevalent in the central-western areas, PopB2 was distributed in central-eastern areas, and PopA2 was limited to an eastern area of RER (Fig. S7).

The measures of genetic diversity for each main population defined by STRUCTURE are summarized in Table 2. PopA and PopB showed a low expected heterozygosity (mean He = 0.258), which was higher than the observed heterozygosity (mean Ho = 0.056). Furthermore, the inbreeding coefficients (Fis) were very high (mean Fis = 0.807).

**TABLE 2:** Population genetic characterization of the *L. infantum* population A and B circulating in the Emilia-Romagna region, northern Italy^*b*^

Population^*a*^	*N*	*P*	MNA	He	Ho	*F*_is_
A	40	0.667	2.867	0.259	0.068	0.739
B	33	0.867	2.600	0.311	0.043	0.864
Mean	36	0.767	2.733	0.285	0.056	0.807

^*a*^ Population, cluster as inferred by STRUCTURE analysis; *N*, sample size; *P*, proportion of polymorphic loci; MNA, mean number of alleles; He, expected heterozygosity; Ho, observed heterozygosity; and *F*_is_, inbreeding coefficient.

^*b*^ Part of the data were published in reference 10.

**Fig 6:**
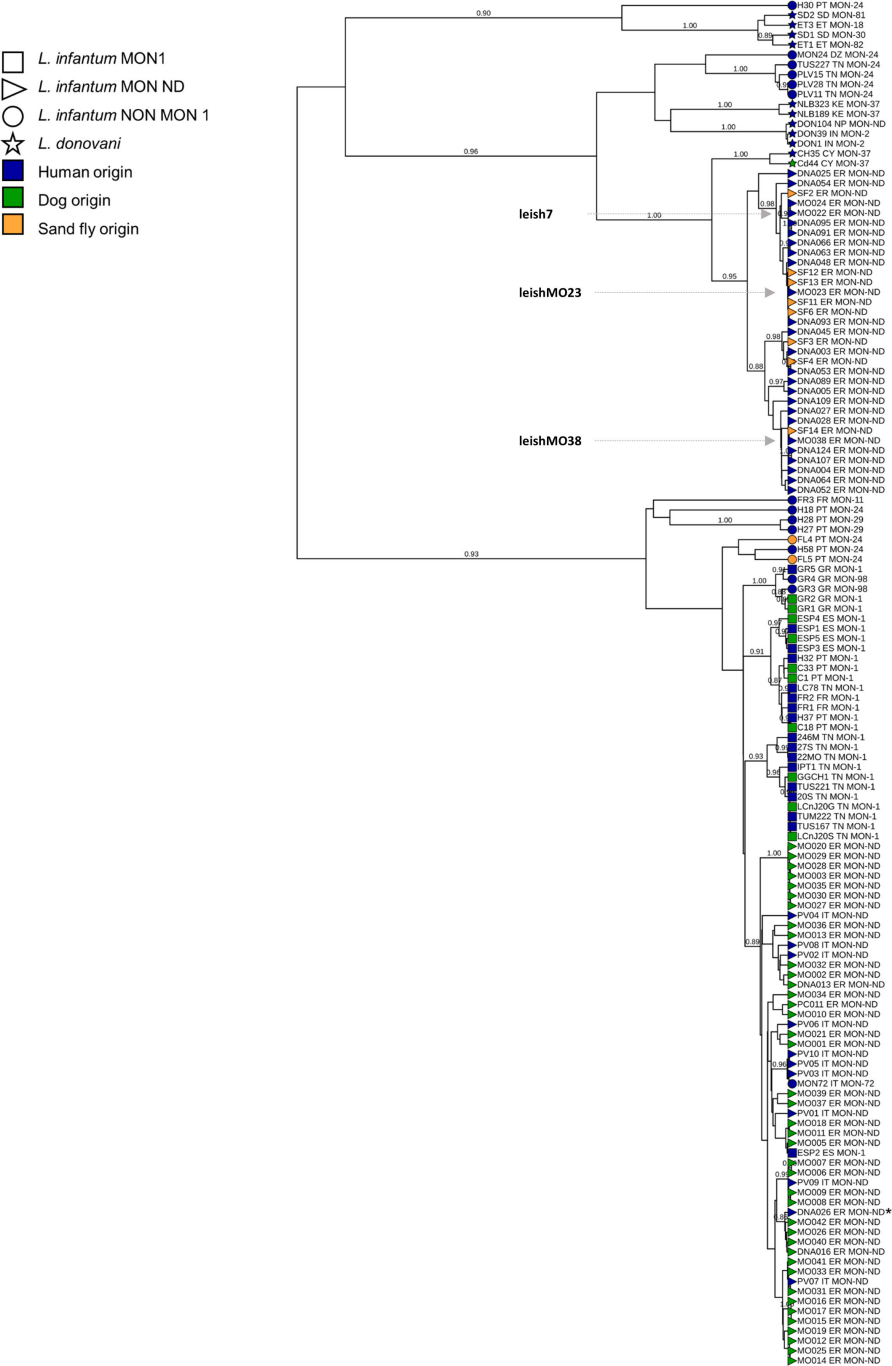
Bayesian phylogenetic tree of 73 *L. infantum* strains from the Emilia-Romagna region, 65 MLMT profiles available in the literature, and 3 WHO reference strains. The tree was obtained by the Sainudiin model using data from 14 coincident microsatellite loci. Posterior probabilities > 0.80 are shown near the nodes. Strain designations specify, respectively, the laboratory code, the zymodeme (MON1 indicates strains belonging to the MON-1 zymodeme, NON MON1 indicates strains that do not belong to the MON1 zymodeme, and MON ND indicates not defined strains), the geographical origin (IT, Italy; ER, Emilia-Romagna region; CY, Cyprus; FR, France; SP, Spain; GR, Greece; PT, Portugal; DZ, Algeria; TN, Tunisia; KE, Kenya; SD, Sudan; ET, Ethiopia; IN, India; and NP, Nepal). The isolates that were sequenced in this study (leish7, leishMO23, and leishMO38) are indicated in bold and bigger font size. Asterisk indicates that the strain was obtained from a VL case that was infected in southern Italy.

The global phylogenetic analysis showing the position of human and sand fly strains from RER within the *L. donovani* complex is represented in Fig. 6. Canine *L. infantum* strains from RER belonging to the original PopA were grouped in a monophyletic clade together with VL strains from other Italian regions and Mediterranean human and canine strains belonging to *L. infantum* zymodemes MON-1, MON-72, and MON-98. On the contrary, *L. infantum* strains from VL patients and sand flies from RER belonging to PopB grouped in a monophyletic clade that was intermediate between *L. infantum* and *L. donovani* clusters. In addition, the PopB clade was close to the cluster, which included two strains from Cyprus, namely, CH35 and CD44cl.1, both classified as *L. donovani* MON-37 (26).

## DISCUSSION

The present study combines comparative genomics with molecular epidemiology approaches to deliver a comprehensive, high-resolution investigation of intraspecific genetic diversity and heterozygosity within the *L. infantum* species in Italy, one of the most important *Leishmania* species worldwide from both a veterinary and public health perspective. We revealed a remarkable heterogeneity in Italian *L. infantum* isolates that are related to various parasite genotypes of different geographic origins, including a hybrid-like genotype first described in isolates from Cyprus.

Our results place Italy at the crossroads of *L. infantum* infection in the Mediterranean and inform on the possible origin of regional sub-populations and their local transmission cycles. We identified a series of *L. infantum* strains from Sardinia and Sicily that are genetically close to those from Spain (southwestern[SW] Europe) and Tunisia (North Africa), highlighting Italy’s central location in the Mediterranean Basin and its historical and current role as one of the main routes of human migration. Based on genetic similarity, the strains leish3 and leish5 likely belong to the North African MON-1 population endemic in southern and eastern Mediterranean regions. This population is genetically different from the European MON-1 population found in SW Europe and South America, which corresponds to the strains leish4 and leish16 and includes the JPCM5 strain used as a reference in this study.

The complex *Leishmania* epidemiology is the result of the parasite’s transmission dynamics, the distribution and geographic abundance of competent vectors, past and current exposure of human populations to the parasite, environmental parameters that impact the seasonal activity and distribution of sand flies (e.g., air temperature, relative humidity, ground cover, altitude, and vegetation), and the presence of reservoir hosts. In Italy, as in the whole Mediterranean basin, dogs (*Canis lupus familiaris*) are incriminated as the primary reservoir hosts of zoonotic human leishmaniasis, even if other domestic and wild mammals infected with *L. infantum* could play a possible role in the biological cycle of the parasite (27). When compared to *L. donovani* species, *L. infantum* MON-1, the most common zymodeme in dogs and humans, has been considered a genomically rather homogeneous population regardless of geographic distribution (15). The parasite’s adaptation to dogs may have been an evolutionary advantage, and the frequent mobility of the canine reservoir may have contributed to the spread of the *L. infantum* MON-1 group (28). However, microsatellite analyses and WGS showed that the MON-1 “core” *L. infantum* clade displays a certain degree of isolation by distance. Three distinct populations have been shown to circulate within the Mediterranean Region, i.e., SW Europe, North Africa, and South-Eastern Europe (15). Thus, the genetic structure within *L. infantum* MON-1 population is mainly related to the parasites’ geographic origin, echoing our previous epidemiological investigation conducted in Tunisia that revealed ecology rather than clinical phenotype as a key driver of parasite genomic adaptation (2).

Our investigation further uncovered putative hybrid strains from RER (leish7, leishMO23, and leishMO38), which may be linked to the re-emergence of VL in this region. Even though human and canine leishmaniasis cases have been mainly reported from central and southern Italy in previous decades, human leishmaniasis is recently spreading in the northern part of the country, including RER. Our MLMT and whole-genome sequencing analyses demonstrated the transmission in RER of putative hybrid populations that are related to divergent *L. infantum* strains first described in Cyprus as judged by the presence of 34,778 shared SNPs (74% of the total SNP count). Previous analyses described the Cypriot strains (e.g., strain CH33) as a novel *L. donovani sensu lato* (s.l.) group based on its unusual MON-37 zymodeme (26, 29), which may either represent a distinct evolutionary lineage within the *L. donovani* complex or an ancient hybrid between *L. infantum* and *L. donovani* (15).

Similar to Cyprus, leishmaniasis in RER displays a peculiar epidemiology. The first documented epidemic of VL dates back to 1971–1972, two decades before the reported northward spread of canine leishmaniasis in Italy. In addition to the extreme summer dryness of 1971, which likely contributed to an excess of sand flies and increased human exposure to their bites, the epidemiologic analysis led to the hypothesis that a cryptic infection in an unidentified domestic or wild animal reservoir was the cause of the outbreak. Following a sharp decline in human leishmaniasis cases in the ensuing decades, RER has seen a re-emergence of VL cases since 2010 (7). We observed that this area is experiencing two distinct *Leishmania* transmission cycles, one of which primarily affects humans involving *Ph. perfiliewi* as a vector (PopB by MLMT analysis) and the other affecting dogs (PopA). In line with a previous study (10), we observed geographic and temporal overlap between these two parasite populations, each showing intraspecific recombination or selfing as indicated by the high *F*_is_ value and low observed heterozygosity. As a hypothesis, the sympatric circulation of distinct *Leishmania* populations could be explained by the involvement of different vectors and/or reservoir species (12) in the transmission dynamics of the parasites. The sand fly fauna of RER is composed of two proven vectors of *Leishmania*, i.e., *Ph. perfiliewi* and *Ph. perniciosus*, with an overwhelming presence of the former in rural areas and the sylvatic environment (30). Further studies are needed to assess the differences in vectorial competence of the two sand fly species as well as the role of wildlife in the epidemiology of the *Leishmania* life cycle in RER.

In conclusion, our genomic and epidemiologic data uncover a remarkable heterogeneity of *L. infantum* isolates in Italy, including a hybrid genotype reminiscent of Cypriot strains. The unusual infection pattern of this putative hybrid highlights the public health risk of introducing new *Leishmania* species due to the increased flow of migrants and tourists from countries where non-European species are endemic (31). The risk of active transmission of newly introduced parasite species may be limited by the lack of competent, local *Phlebotomus* species and/or the absence of permissive reservoir hosts. However, the requirement of animal reservoirs does not apply to parasite species associated with an anthroponotic cycle of transmission, such as *L. tropica* and *L. donovani* (31). In addition, *Leishmania* has a remarkable adaptation capacity to highly diverse ecologies, as illustrated by the introduction of *L. infantum* from the Old World to the New World during the South American conquest, which led to important epidemiological consequences. The challenge of future studies lies in the application of a trans-disciplinary approach combining genomics, epidemiology, ecology, and entomology investigations to identify distinct transmission cycles and the underlying environmental parameters that drive the evolution of distinct, region-specific *L. infantum* sub-populations with unique clinical features.

## MATERIALS AND METHODS

### Clinical samples

Ten isolates of *L. infantum* were obtained from the cryobank of the National Reference Laboratory for Leishmaniasis (C.Re.Na.L., Palermo, Sicily). The strains were originally obtained from different hosts and geographies, including (i) from two naturally infected dogs by lymph node aspiration (MCAN/IT/2021/51327, Sardinia, referred to as leish3; MCAN/IT/2020/1265, Sicily, referred to as leish4), (ii) from the spleen of a wild marten (MMST/IT/2006/V2921, Sicily, referred to as leish5), (iii) from four owned cats by lymph node aspiration (MFEL/IT/2018/10816, Sicily, referred to as leish16; MFEL/IT/2018/CRENAL791; MFEL/IT/2018/CRENALBORIS; MFEL/IT/2018/CRENAL2147), and (iv) from bone marrow aspirates of three human VL cases from RER (MHOM/IT/2014/IZSLER-MO22, referred to as leish7; MHOM/IT/2014/IZSLER-MO23, referred to as leishMO23; MHOM/IT/2016/IZSLER-MO38, referred to as leishMO38). The strains leish3, leish4, leish5, leish7, leish16, leishMO23, and leishMO38 were used for whole-genome sequencing analysis as described in Table 1. A detailed description of parasite culture, DNA extraction, and WGS protocol is provided in the supplemental material.

### Computational analyses

WGS reads were mapped, and sequencing data were processed using GIP version 1.1.0 (1) (see Data Set S1E for mapping metrics). The outputs of the GIP pipeline were used for downstream analyses, ignoring the reads mapping to mitochondrial DNA (i.e., maxi and minicircles). Mean coverage in 300 bp bins as generated by the GIP pipeline was used to compute somy scores per chromosome by first normalizing bin scores for a sample by their median across the entire genome (to obtain comparable values between samples) and then multiplying by two (to scale somy values to the default diploid state assumed for most of the chromosome). These somy scores were represented on “per chromosome and per sample” boxplots (Fig. S1). The median somy scores across bins belonging to a given chromosome were visualized on a sample versus chromosome heatmap (Fig. 1B).

Normalized mean coverages per gene are reported by GIP, as the mean coverage of the gene divided by the median coverage of the chromosome containing the gene. These values were plotted against gene indices, the index of a gene corresponding to its rank when sorted by genomic coordinates (Fig. 2A). These values were also used to compute a heatmap (Fig. 2B). In order to preserve a readable color scale, genes having a normalized mean coverage below 0.5 or above 1.5 in at least one of the samples were not included in the heatmap.

SNP frequencies were retrieved from the filtered output of GIP for each sample. For a given set of samples, the union of all SNPs across samples was considered, assuming an alt-allele frequency of 0 when an SNP was missing from a given sample. Distributions of alt-allele frequencies were represented using violin plots (Fig. 3B). Upset plots (32) (Fig. 3A and 4B) were generated using the upsetplot 0.6.0 Python library (33), considering SNPs at alt-allele frequency 0 as missing from a given sample. SNPs were sorted according to genomic coordinates and assigned a corresponding index against which alt-allele frequencies were plotted (Fig. 3D; Fig. S4). Euclidean distances between samples in the SNP allele frequencies space were computed and used to build a dendrogram using the scipy.cluster.hierarchy module from the Scipy 1.8.0 Python library (Fig. 4A). The clustering was obtained using the UPGMA method.

Besides the more specialized Python libraries indicated above, post-GIP analyses were carried out using the Pandas 1.4.2 (34, 35); Matplotlib 3.5.1 (36); and Seaborn 0.11.2 (37) Python libraries.

Taxonomic assignation of raw Illumina reads for some of the samples was performed using Kraken 2.1.3 (25) against the “protozoa” database in order to get insights into the possible hybridization events at the origin of putative hybrid samples. The summarized taxonomic assignations were extracted from the reports generated by Kraken and represented using radar plots (Fig. 4C).

### Real-time quantitative PCR analysis

Copy number quantification for chr 8 was assessed by qPCR analysis using the SYBR Green assay for the samples leish16 and leish3 and additional feline *Leishmania* strains (see above), as described in the supplemental material. The approach was based on a comparative quantitative detection between a first target, the histone deacetylase (HD) gene on chr 8, and a second target on diploid chr 28 used as a control, corresponding to a gene encoding for a putative glycerophosphoryl diester phosphodiesterase (GDP). Briefly, total genomic DNA was extracted from 1 × 10^6^ parasites using DNeasy Blood & Tissue kit (Qiagen) following the manufacturer’s protocol. qPCR was performed in triplicates and carried out in 20  µL of reaction mixture containing 10 µL of Fast SYBR Green Master Mix (Biorad), 0·2  µL (10 pmol/µL) of gene-specific forward and reverse primers (HD-2F TGTAGCAGCATGCGCGCGT and HD-2R CTAGACGCCGGGGCAATGA, generating a product of 475 bp; GDP-2F GAGCACTTCACTCAA GAGGC and GDP-2R TCGTCGCTCTGTCAGCTGG, generating a product of 466 bp), 2  µL of genomic DNA template, and 7·6 µL nuclease free water to adjust the reaction volume. Negative controls were included in all assays corresponding to the reaction mixture without genomic DNA. Real-time PCR was carried out in QuantStudio 3 Real-Time PCR System, 96-well (Life Technologies, Carlsbad, USA). The following thermal profile was used: pretreatment at 98°C for 2 min, then 40 cycles of 98°C for 5 s, 60°C for 5 s, and a melting curve analysis to verify the amplification product.

### Microsatellite analysis of *Leishmania* samples from RER

The three *L. infantum* isolates from RER (leish7, leishMO23, and leishMO38, see above) have been previously characterized by multilocus microsatellite typing using a panel of 15 dinucleotide microsatellite markers (Li41-56, Li46-67, Li21-34, Li22-35, Li23-41, Lm2TG, Lm4TA, Li71-5/2, LIST7039, Li71-33, Li71-7, CS20, Li45-24, TubCA, and LIST7031) (10). They were representative of a distinct genetic population of *L. infantum* circulating in humans and sand flies but not in dogs, defined as population (Pop) B in Rugna et al. (10). In the present study, we expanded the epidemiologic characterization of the *L. infantum* strains circulating in RER by comparing their MLMT profiles to those of 30 *L*. *infantum* strains from VL (*N* = 22) and sand flies (*N* = 8) (Data Set S2A) and 40 strains from dogs (10) (Data Set S2B), for a total of 73 strains obtained from various areas of RER between 2013 and 2021.

MLMT profile results were analyzed using models based on the Bayesian clustering algorithm and genetic distances (supplemental material). Moreover, descriptive statistics for genetic populations and phylogenetic analyses were performed as described in the supplemental material.

A phylogenetic tree based on 14 coincident markers was created as described in the supplemental material for a global microsatellite analysis, and it included the MLMT profiles of the 73 strains from ER and those of 65 strains of the *L. donovani* complex, including (i) 11 strains from other endemic Italian regions (10) (Data Set S1B), (ii) 51 strains from other countries (Data Set S2C), and (iii) three WHO *L. infantum* reference strains, namely, MHOM/TN/80/IPT1 (MON-1), MHOM/IT/86/ISS218 (MON-72), and MHOM/DZ/82/LIPA59 (MON-24) (Data Set S2A), which represent the most common zymodemes responsible for leishmaniasis in Italy.

## Data Availability

All relevant data are within the paper and its supplemental files. Genome sequence data have been deposited in BioProject at PRJNA1008390.

## SUPPLEMENTAL MATERIAL

Data Set S1 mbio.00995-24-s0001.xlsx WGS supporting information.

Data Set S2 mbio.00995-24-s0002.xlsx MLMT supporting information.

Supplemental material mbio.00995-24-s0003.docx Supporting methods and supplemental figures.

ASM does not own the copyrights to Supplemental Material that may be linked to, or accessed through, an article. The authors have granted ASM a non-exclusive, world-wide license to publish the Supplemental Material files. Please contact the corresponding author directly for reuse.
