# Supplementary material for: Genomic and epidemiological evidence for the emergence of a L. infantum/L. donovani hybrid with unusual epidemiology in northern Italy
Source: mBio. 2024 Jun 4;15(7):e00995-24. doi: 10.1128/mbio.00995-24 (PMC11253594; doi:10.1128/mbio.00995-24)
Supplement: Supplemental material — Supporting methods and supplemental figures. [file mbio.00995-24-s0003.docx]

**Supporting Information for**

**Genomic and epidemiological evidence for the emergence of a *L. donovani/L. infantum* hybrid with unusual epidemiology in Northern Italy**

Bruno F. ^1†^, Castelli G. ^1†^, Li B. ^7^, Reale S. ^1^, Carra E. ^2^, Vitale F. ^1^, Scibetta S. ^1^, Calzolari M. ^2^, Varani S. ^3^, Ortalli M. ^3,4^, Franceschini E. ^5^, Gennari W. ^6^, Rugna G. ^2‡^ and G.F. Späth ^8‡^

^1^Centro di Referenza Nazionale per le Leishmaniosi (C.Re.Na.L.), WOAH Leishmania Reference Laboratory, Istituto Zooprofilattico Sperimentale della Sicilia, Via Gino Marinuzzi 3, 90129 Palermo, Italy; ^2^ Istituto Zooprofilattico Sperimentale della Lombardia e dell'Emilia Romagna "B. Ubertini", via A. Bianchi 9, 25124 Brescia, Italy; ^3^University of Bologna, Department of Medical and Surgical Sciences, 40138 Bologna, Italy; ^4^IRCCS Azienda Ospedaliero-Universitaria di Bologna, 40138 Bologna, Italy; ^5^Infectious Disease Unit, Azienda Ospedaliera Universitaria di Modena, Largo del Pozzo 71, 41124, Modena, Italy; ^6^Virology and Molecular Microbiology Unit, University Hospital of Modena, Modena, Italy; ^7^Institut Pasteur, Université Paris Cité, Bioinformatics and Biostatistics Hub, F-75015 Paris, France; ^8^Institut Pasteur, Université Paris Cité, INSERM U1201, Unité de Parasitologie moléculaire et Signalisation, F-75015 Paris, France.

^‡^co-corresponding authors: Gerald Späth, [gerald.spaeth@pasteur.fr](mailto:gerald.spaeth@pasteur.fr); Gianluca Rugna [gianluca.rugna@izsler.it](mailto:gianluca.rugna@izsler.it)

**This PDF file includes:**

Supporting text

Supplementary Figures S1 to S7 and figure legends

Legends for Datasets S1 to S2

SI References

**Other supporting materials for this manuscript include the following:**

Datasets S1 to S2 (separate files)

Supporting Information text

**Methods**

**Parasite culture and DNA extraction**. Parasites were grown in Evans’ modified Tobie’s medium (EMTM) supplemented with 10% fetal bovine serum, 5% sterile human urine, 1% phenol red solution, and 2% antibiotic solution (250 mg/ml gentamicin, and 500 mg/ml 5-fluorocytosine (1). At day seven, parasite viability and growth were assessed microscopically, and cells were used to inoculate 10 ml of RPMI-PY medium (1). After reaching exponential growth phase (approx. 5 days), cells were pelleted by centrifugation at 2100 g for 10 min at 4 °C, and washed three times with physiological solution (0.9% NaCl) prior to DNA extraction according to a modified protocol described in Castelli *et al.* (2020) (2). Briefly, cells were lysed by heat (96˚C for 20 minutes) with 400 μl of a mixture containing 20% Chelex resin (Sigma, St. Louis, MO, USA), 1% Nonidet P-40 (Sigma, St. Louis, MO, USA), 1% Tween 20 (Sigma, St. Louis, MO, USA), and sterile distilled water. The mixture was centrifuged at 14,000 g for 10 minutes at 4˚C and the DNA-containing, upper phase was collected and stored at -20˚C(3). For multilocus microsatellite typing, DNA from sand fly samples was extracted using Qiagen DNeasy Blood & Tissue Kit (Qiagen, Hilden, Germany) according to the manufacturer’s instructions, and eluted in a final volume of 100 μl of elution buffer. DNA of strains isolated from VL cases was extracted from 100 μl whole blood (peripheral blood) or 100 μl bone marrow aspirate using NucliSENSeasyMAG (bioMerieux, Marcy l’Etoile, France).

**Genome sequencing.** Whole genome DNA library preparation was performed using 100 ng DNA as input according to the standard protocol for Nextera DNA Flex Library Prep (Illumina, CA, USA) using Nextera DNA CD Indexes (Reference Guide 1000000025416.v07, Illumina). Quality and fragment size of all libraries were assessed by capillary electrophoresis on a TapeStation 4200, using a High Sensitivity DNA kit (Agilent, CA, USA). Individual DNA libraries were quantified using Qubit 2.0 Fluorometer with High Sensitivity dsDNA Assay (Thermo Fisher Scientific, MA, USA), and diluted to 4 nM with Resuspension Buffer. Normalized libraries were pooled using 10 μl per sample prior to sequencing, then the pooled library was diluted to a final concentration of 12 pM and denatured according to the sequencing protocol for V3 cartridges used with the Illumina MiSeq platform (Reference Guide 15039740.v10, Illumina). Samples were spiked with 1% of PhiX Control library and sequencing was run using a paired-end 300-cycle protocol.

**Real time quantitative (q) PCR analysis.** Total genomic DNA was extracted from 1x10^6^ parasites using DNeasy Blood & Tissue kit (Qiagen) following the manufacturer’s protocol. qPCR was performed in triplicates and carried out in 20 µl of reaction mixture containing 10 µl of Fast SYBR® Green Master Mix (Biorad), 0.2 µl (10 pmol/µl) of gene-specific forward and reverse primers (HD-2F TGTAGCAGCATGCGCGCGT and HD-2R CTAGACGCCGGGGCAATGA, generating a product of 475bp; GDP-2F GAGCACTTCACTCAA GAGGC and GDP-2R TCGTCGCTCTGTCAGCTGG, generating a product of 466bp), 2 µl of genomic DNA template and 7.6 µl nuclease free water to adjust the reaction volume. Negative controls (NTC) were included in all assays corresponding to reaction mixture without genomic DNA. Real-Time qPCR was carried out in QuantStudio™ 3 Real-Time PCR System, 96-well (Life Technologies, Carlsbad, USA). The following thermal profile was used: pretreatment at 98 °C for 2 min then 40 cycles of 98 °C for 5 sec, 60 °C for 5 sec and a melting curve analysis to verify the amplification product.

**Population structure and phylogenesis based on MLMT.** Clustering analysis was performed by STRUCTURE v.2.3.4 software package (4), which determines genetically distinct populations on the basis of allele frequencies and estimates the individual membership coefficient (Q-value) in each probabilistic population. The Markov chain Monte Carlo iterations were set to 200,000 and the length of burn-in period to 20,000. For each value of K (estimated number of populations) between K=1 and K=10, ten independent simulations were performed. To estimate the best number of populations, the likelihood values computed by STRUCTURE were analyzed by STRUCTURE HARVESTER v0.6.1 (5) implementing the Evanno’s ΔK statistic, which is based on the rate of change in the log probability of data between successive K values (6). Data were sorted by CLUMPP 1.1.2 software (7) and the barplots of the CLUMPP outfiles were visualized using an online tool, STRUCTURE PLOT (8). Descriptive statistics for genetic populations were calculated by the GDA 1.1 software (9); this included mean number of alleles (MNA) per population, proportion of polymorphic loci (P), expected (He) and observed (Ho) heterozygosity and inbreeding coefficient (Fis). Phylogenetic analysis was performed based on microsatellite genetic distances using the application BEASTvntr package implemented in the BEAST2. 2.4.3 (10). The diploid data were entered as two distinct partitions, with a linked tree and strict clock; the Sainudiin mutation model of microsatellites (11) was then selected with a chain length of ten million steps. Trees were visualized with iTOL v6 (https://itol.embl.de/).


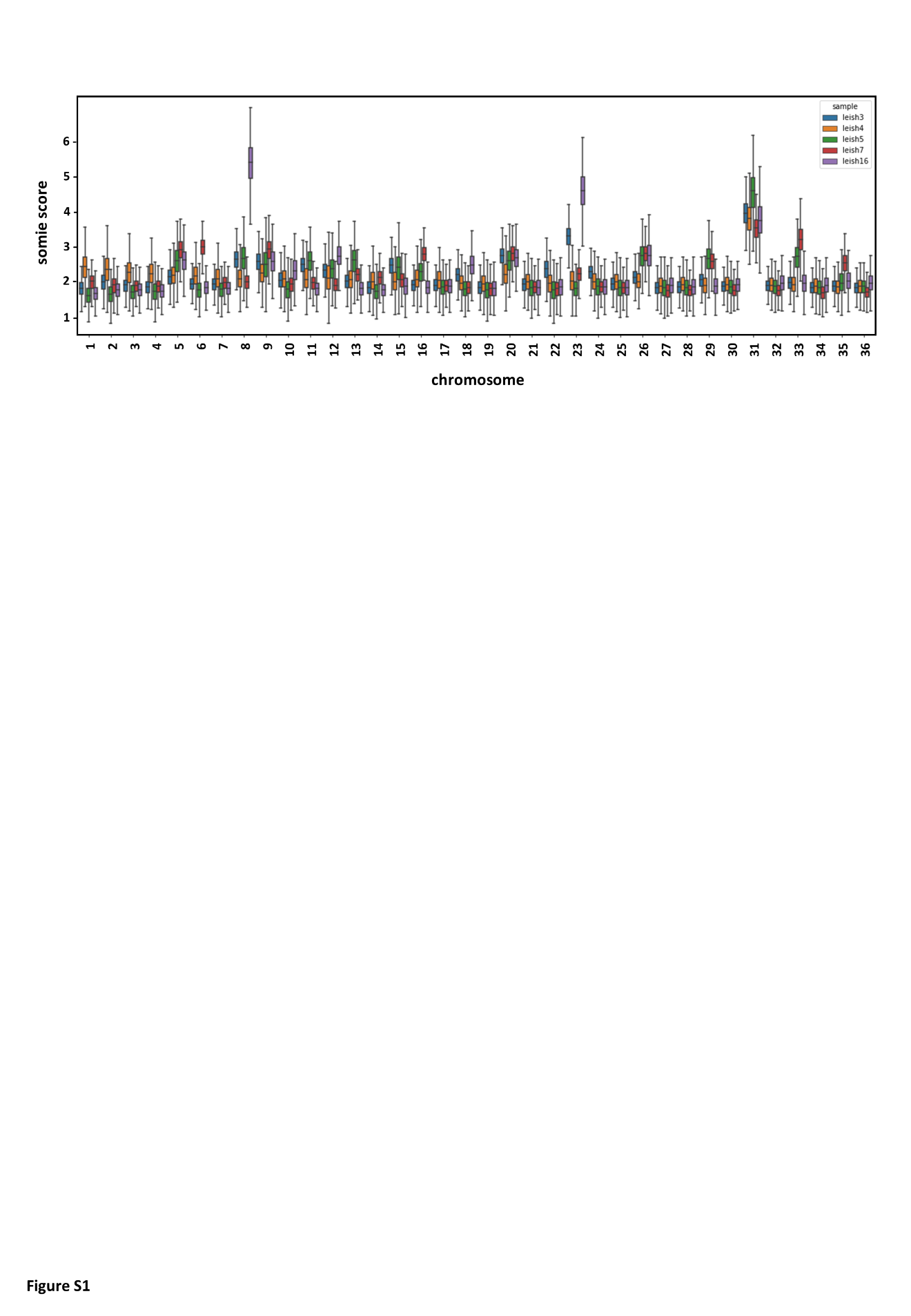


Figure S1. Box plot representing the normalized sequencing coverage distributions for each chromosome (X-axis) expressed as somy score (Y-axis). The horizontal line in each box indicates the median normalized coverage value. The lower and upper edges of the box show respectively the lower quartile and upper quartile of normalized coverage values. The whiskers show maximum and minimum coverage values excluding outliers, which are not shown to ease readability. Different strains are shown in different colors (see legend in the figure).


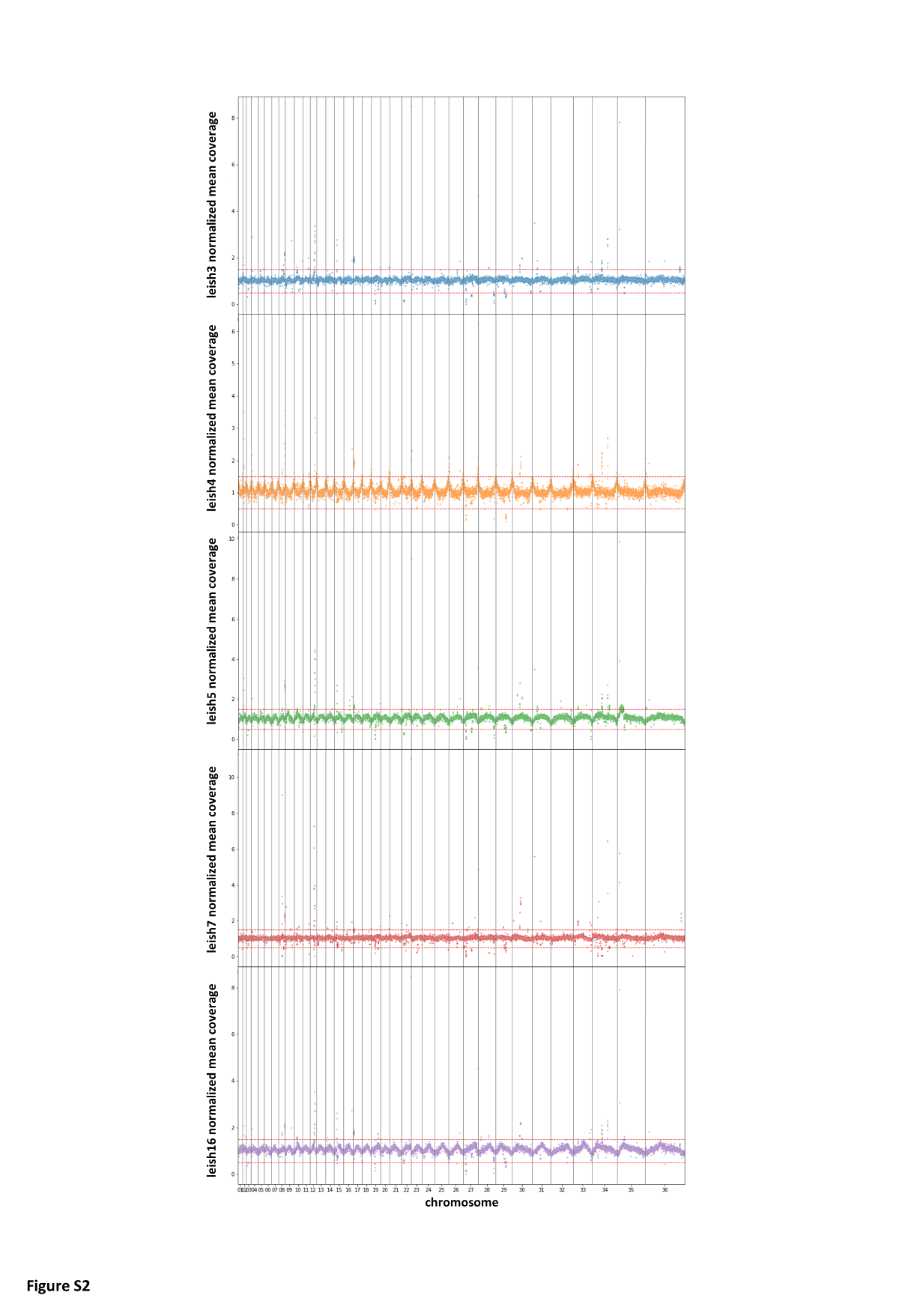


Figure S2. Scatterplots showing gene CNVs for the individual strains revealed by differences in normalized mean coverage. The X-axis represent genomic indices (their rank when sorted by genomic coordinates of their start positions). The Y-axis represents the genes’ normalized mean coverage (see methods).


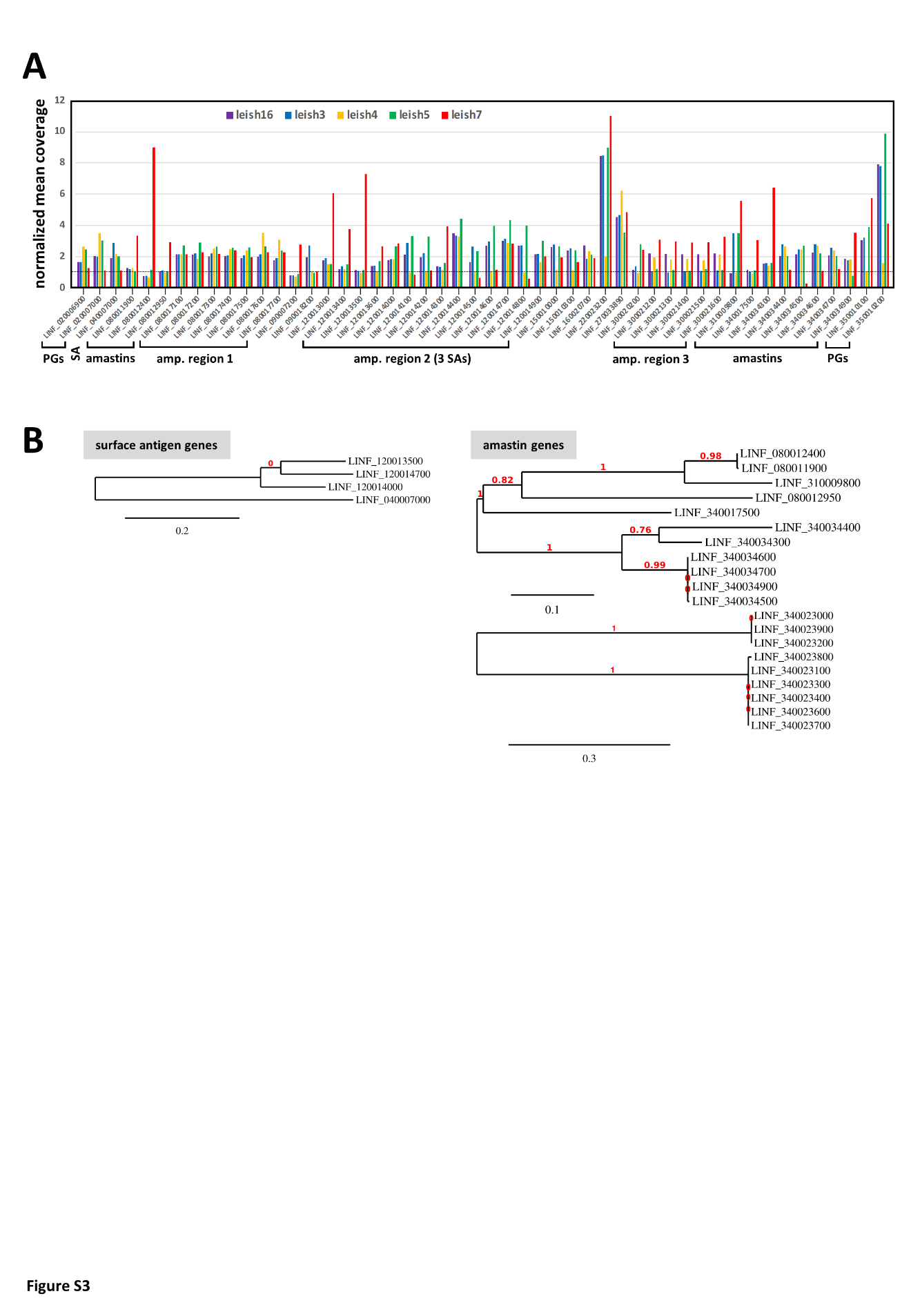


Figure S3. (A) Convergent amplifications revealed by similar coverage changes (Y-axis) for the genes indicated (X-axis) across the analysed strains (see colour code). (B) Phylogenetic analysis of the indicated genes performed on the www.phylogeny.fr web service using FASTA-formatted sequence files.

**
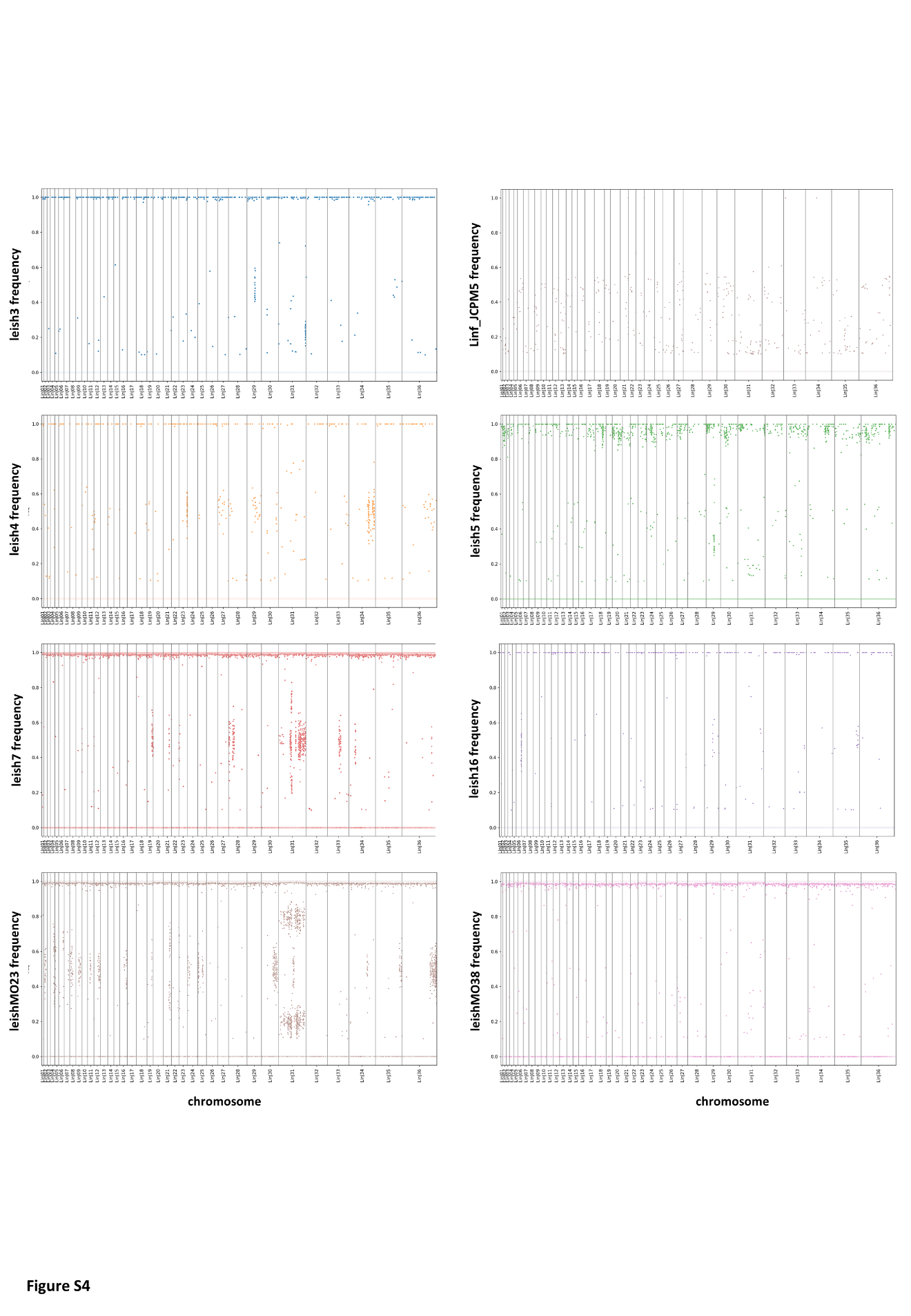
**

**Figure S4.** SNP scatterplots for the indicated strains (see Y-axis labeling). The Y-axis represents the SNPs genomic index (rank when sorted by genomic position) and the X-axis represents their frequency.

**
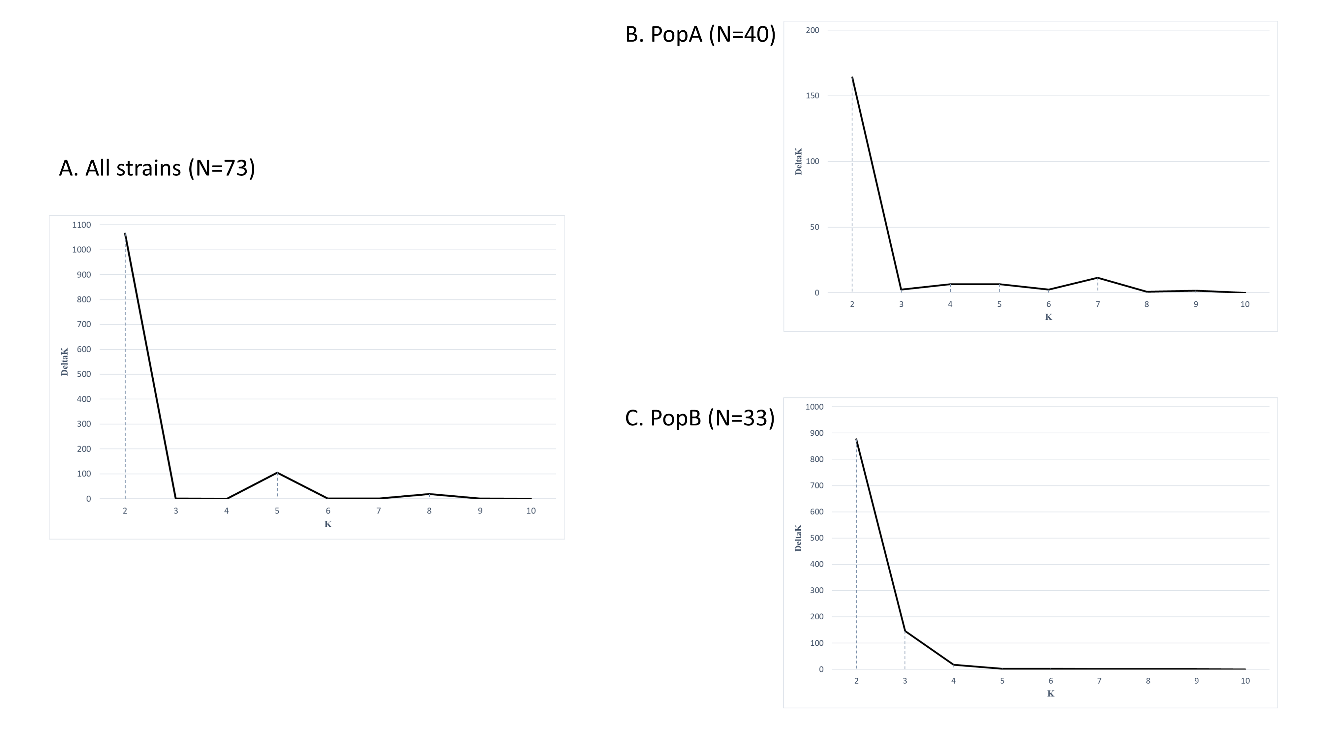
**

**Figure S5.** Plot of the ΔK values per populations (K) according to the Evanno method on data generated by STRUCTURE analysis performed on: (A) all strains, (B) Population A, (C) Population B. The primary peaks indicate the most probable number of populations.


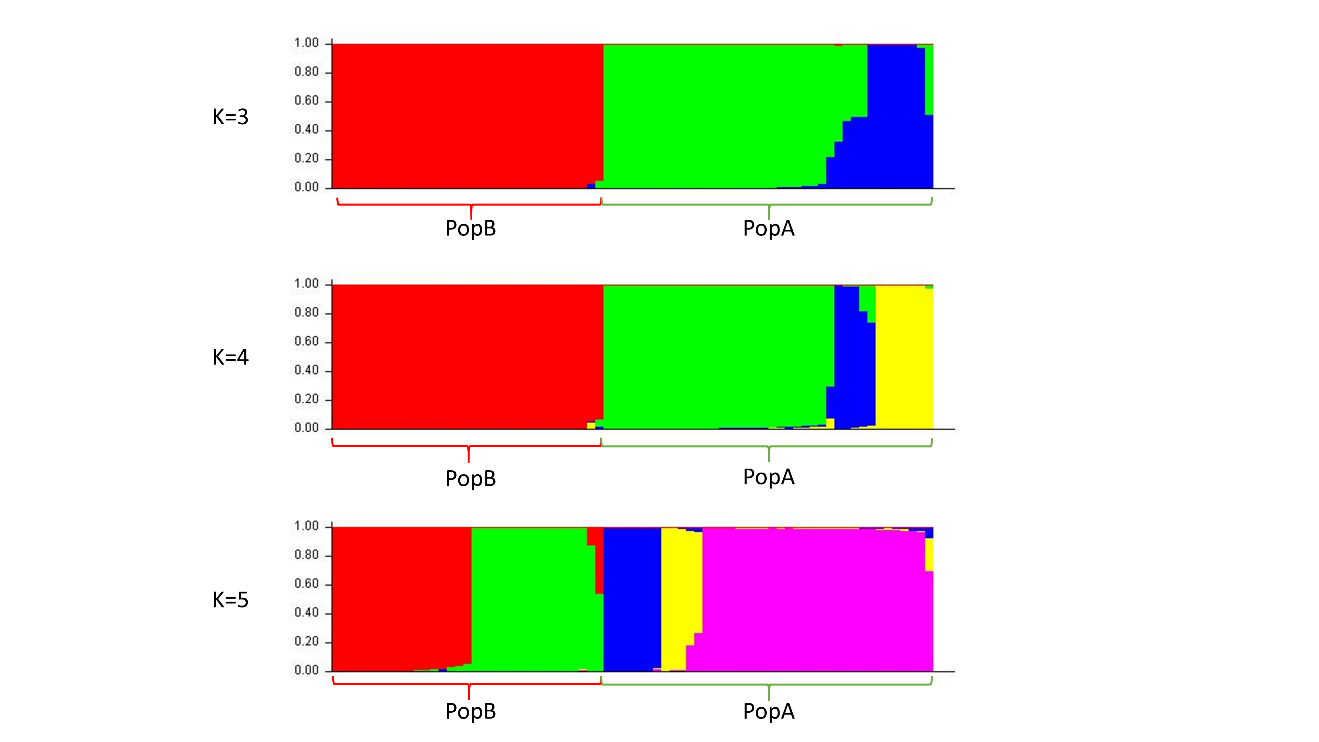


**Figure S6.** Population structure of the 73 *Leishmania* strains from the Emilia-Romagna region determined by STRUCTURE. Averages of 10 STRUCTURE runs at each value of K from 3 to 5 where each strain is represented by a single vertical line divided into K colours, K being the assumed number of clusters, with each colour representing one cluster and the length of the coloured segment indicating the individual strain’s estimated degree of kinship to that cluster.


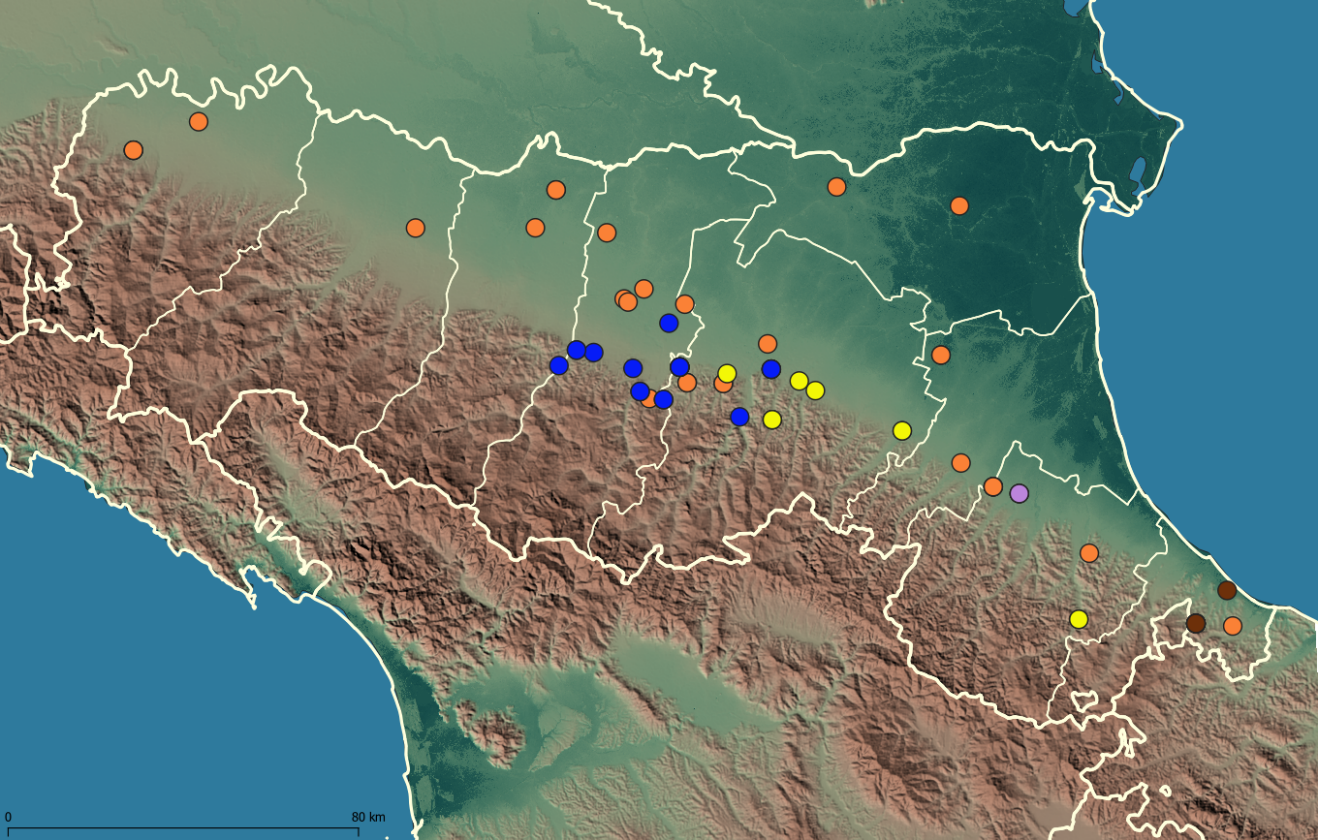


**Figure S7.** Geographical distribution of human, canine and sand fly *Leishmania*-positive samples, 2013–2021, Emilia-Romagna region (northern Italy). Colors depict the subpopulations inferred by STRUCTURE analysis: orange for PopA1; brown for PopA2; yellow for PopB1; blue for PopB2. Map generated with Quantum-GIS (<https://www.qgis.org/it/site/>).

Datasets legends S1 to S2 (separate files).

**Dataset S1A:** All gene CNVs

**Dataset S1B:** Most significant gene CNVs

**Dataset S1C:** Gene depletions

**Dataset S1D and S1Dbis:** SNP frequencies

**Dataset S1E:** Mapping metrics

**Dataset S2A**: Designation, characteristics and MLMT profiles of the *Leishmania* strains

**Dataset S2B:** Designation and characteristics of Italian *Leishmania* strains used for comparison

**Dataset S2C**: *Leishmania donovani* complex strains used in this study for comparison.

SI References

1. Castelli G, Oliveri E, Valenza V, Giardina S, Facciponte F, La Russa F, Vitale F, Bruno F. 2023. Cultivation of Protozoa Parasites In Vitro: Growth Potential in Conventional Culture Media versus RPMI-PY Medium. Vet Sci 10:252.
2. Castelli G, Bruno F, Caputo V, Fiorella S, Sammarco I, Lupo T, Migliazzo A, Vitale F, Reale S. 2020. Genetic tools discriminate strains of Leishmania infantum isolated from humans and dogs in Sicily, Italy. PLoS Neglected Tropical Diseases 14:e0008465.
3. Bulle B, Millon L, Bart J, Gallego M, Gambarelli F, Portus M, Schnur L, Jaffe CL, Fernandez-Barredo S, Alunda JM, Piarroux R. 2002. Practical approach for typing strains of Leishmania infantum by microsatellite analysis. J Clin Microbiol 40:3391-3397.
4. Pritchard JK, Stephens M, Donnelly P. 2000. Inference of population structure using multilocus genotype data. Genetics 155:945-959.
5. Earl DA, VonHoldt BM. 2012. STRUCTURE HARVESTER: a website and program for visualizing STRUCTURE output and implementing the Evanno method. Conservation genetics resources 4:359-361.
6. Evanno G, Regnaut S, Goudet J. 2005. Detecting the number of clusters of individuals using the software STRUCTURE: a simulation study. Mol Ecol 14:2611-2620.
7. Jakobsson M, Rosenberg NA. 2007. CLUMPP: a cluster matching and permutation program for dealing with label switching and multimodality in analysis of population structure. Bioinformatics 23:1801-1806.
8. Ramasamy RK, Ramasamy S, Bindroo BB, Naik VG. 2014. STRUCTURE PLOT: a program for drawing elegant STRUCTURE bar plots in user friendly interface. SpringerPlus 3:1-3.
9. Lewis PO, Zaykin D. 2012. Genetic Data Analysis: computer program for the analysis of allelic data, Version 1.0 (d16c), 2001. Free Program Distributed by the Authors over the Internet
10. Bouckaert R, Heled J, Kühnert D, Vaughan T, Wu C, Xie D, Suchard MA, Rambaut A, Drummond AJ. 2014. BEAST 2: a software platform for Bayesian evolutionary analysis. PLoS computational biology 10:e1003537.
11. Sainudiin R, Durrett RT, Aquadro CF, Nielsen R. 2004. Microsatellite mutation models: insights from a comparison of humans and chimpanzees. Genetics 168:383-395.
